# Exophthalmos as a Presenting Manifestation of Limited Wegener’s Granulomatosis in a Patient with Prior Graves’ Disease

**DOI:** 10.4137/ccrep.s731

**Published:** 2009-06-30

**Authors:** Brian Bowers, Deepak Gupta, Hirenkumar Patel, Naureen Mirza

**Affiliations:** Department of Medicine, Division of Rheumatology, Stony Brook University Medical Center, T16-040 Health Science Center, Stony Brook, N.Y. 11794. Email: bpbowers@optonline.net

**Keywords:** Limited Wegener’s granulomatosis, ocular, orbit, vasculitis, exophthalmos

## Abstract

Wegener’s granulomatosis is a granulomatous necrotizing vasculitis characterized by its predilection to affect the upper and lower respiratory tracts and kidneys. Ocular manifestations such as keratitis, conjunctivitis, scleritis, episcleritis, nasolacrimal duct obstruction, uveitis, retroorbital pseudotumor with proptosis retinal vessel occlusion, and optic neuritis have all been described. We present a case of limited Wegener’s granulomatosis presenting with proptosis.

A 57 year old woman with a history of Graves’ disease 20 yrs ago, presented to her ophthalmologist with new onset of exophthalmos of right eye. An MRI of the brain and orbits revealed a soft tissue mass behind the right orbit. The biopsy of the mass revealed transmural inflammation with fibrinoid necrosis consistent with necrotizing vasculitis. She was diagnosed with Wegener’s granulomatosis limited to the eye and was treated with oral cyclophosphamide and prednisone followed by weekly methotrexate with good response.

Though ocular manifestations of Wegener’s granulomatosis are well described, a review of the literature revealed that exophthalmos as the lone presenting manifestation is quite rare. Only two cases have been reported in the English literature since 1977. This case illustrates the importance of considering a diagnosis of limited Wegener’s granulomatosis presenting with proptosis of the orbit.

## Introduction

Wegener’s Granulomatosis (WG) is a severe, granulomatous necrotizing vasculitis which classically affects the upper and lower tracts and kidneys. Limited forms of WG have been described which spares the kidneys. It has been found to have a less severe course and a good response to cytotoxic therapy. Though ocular manifestations of WG are common, a review of literature found only two cases in which exophthalmos was the presenting feature. We present a case of a woman with history of Graves’ disease presenting with exophthalmos that was found to have a limited form of WG.

## Case Report

**Figure 1:**
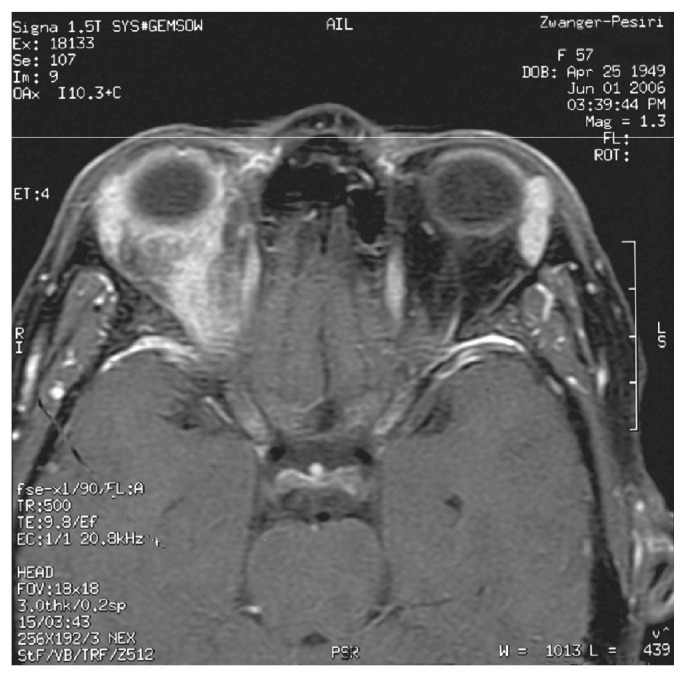
MRI of brain and orbit which reveals enhancement of soft tissue intimately associated with right optic nerve sheath.

**Figure 2:**
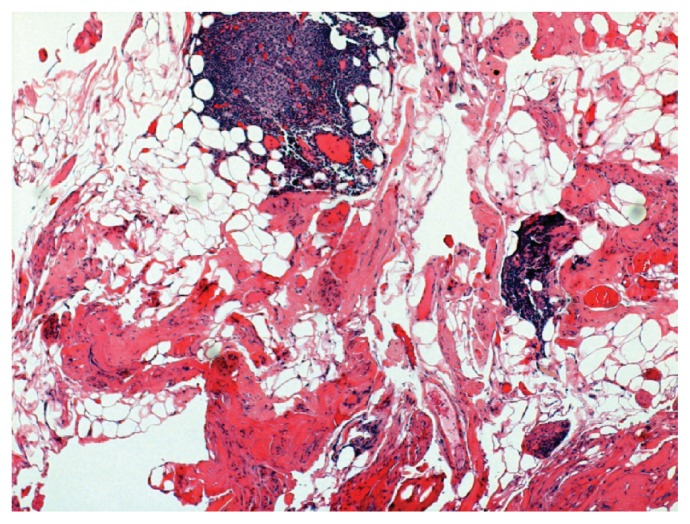
Transmural inflammation with fibrinoid necrosis consistent with Limited Wegener’s Granulomatosis. There is also evidence of lymphocytic infiltration in the fatty tissue consistent with Grave’s Disease.

A 57 year old woman presented to her ophthalmologist complaining of protrusion of her right eye in August 2005. She had exophthalmos of left eye 20 years ago and she was diagnosed as Graves’ disease which resolved without any treatment or intervention. She was without complaint for 20 years until presenting to her ophthalmologist who felt that her exophthalmos was due to Graves’ disease but she was found to be borderline hypothyroid. Six months later, she developed redness, dryness, and pain in the right eye with worsening exophthalmos. She also complained of double vision and headaches. She was started on thyroid replacement for borderline hypothyroidism but had no relief in proptosis. Antibiotics were prescribed for suspected bacterial infection of the right eye without resolution of her symptoms. She was then started on prednisone 80 mg daily for possible autoimmune/inflammatory etiology and her symptoms improved significantly. At this time, her visual acuity was noted to be 20/25 in the affected eye. Fundoscopic exam was normal. Hertel exophthalmometry, ocular tension, and biomicroscopy were not documented. An MRI of brain and orbit revealed an enhancing soft tissue mass behind the right globe (Fig. 1). The biopsy of the soft tissue mass revealed transmural inflammation with fibrinoid necrosis consistent with necrotizing vasculitis as well as also evidence of lymphocytic infiltration in the fatty tissue consistent with Graves’ disease (Fig. 2). ANA, ANCA, ACE, anti-SSA and anti-SSB antibodies were negative though the patient was found to have an equivocal MPO-ANCA of 6. A diagnosis of limited WG was made. Patient was started on oral cyclophosphamide at 2 mg/kg/day in August 2006 and prednisone was continued. A repeat MRI of the orbit in November 2006 showed decreased enhancement and infiltration of fat adjacent to right optic nerve. In April 2007, cyclophosphamide was discontinued because of leucopenia and methotrexate was started. The prednisone has been discontinued. She continues to remain stable with no worsening till to date.

## Discussion

WG is a granulomatous necrotizing vasculitis characterized by its predilection for the upper and lower respiratory tracts and in most cases, the kidneys.^1^ Cutaneous, musculoskeletal, gastrointestinal, and cardiac manifestations have been reported. Ocular manifestations include keratitis, conjunctivitis, scleritis, episcleritis, nasolacrimal duct obstruction, uveitis, and optic neuritis and have been reported in up to 58% of patients with WG.^2^

Our case is interesting as this woman presented with exophthalmos only. This finding presented a diagnostic conundrum due to the patient’s prior history of Graves’ disease. Ocular complaints have been reported as the initial symptom in 8 to 16% of WG. A review of the literature found only 2 cases in which proptosis/exophthalmoswasthepresentingclinicalmanifestation.^2^,^3^ Proptosis, in regards to WG, is a common finding as it has been reported in up to 22% of all cases with ocular manifestations of WG.^3^ ANCA titers are also not helpful in this setting as they are usually not elevated in limited WG. An association between patients with hyperthyroidism treated with antithyroid medications and the development of ANCA associated vasculitis has been reported in the literature.^7^ The prevalence of the Grave’s disease and limited Wegener’s Granulomatosis occurring in the same patient is unknown.

Biopsies in WG primarily yield inflammatory lesions which include necrosis, granulomatous changes, and vasculitis. The diagnostic yield is dependent on the site and type of tissue biopsied. Tissue from transbronchial biopsies have been shown to be diagnostic in <7% whereas open lung biopsies have been found to be diagnostic in >90%.^4^ It has been reported that orbital biopsies rarely show the classic triad of vasculitis, tissue necrosis, and granulomatous inflammation.^5^ A majority of patients in this review were found to have vasculitis with either necrosis or granulomatous change.^5^ In our case, 2 of 3 features were present as transmural inflammation with fibrinoid necrosis. The biopsy also revealed lymphocytic infiltration of the retroorbital adipose tissue suggestive of Graves’ disease.

The advent of combination therapy such as cyclophosphamide and steroids has significantly dropped mortality rates in systemic WG. Ocular manifestations of WG can cause significant and, at times, irreversible damage. Visual morbidity has been reported in 8%–17% of cases and significant visual loss has been reported in up to half the patients.^6^ Thus prompt diagnosis and early intervention can reduce the incidence of ocular damage.

## Conclusion

Severe ocular morbidity may be a complication in both limited and severe forms of WG. The signs and symptoms may be nonspecific or as in our case may mimic other illnesses. The goal of presenting this case is to expose practitioners to the various ocular manifestations of limited WG to aid in early diagnosis and treatment.
